# Paraspinal extramedullary hematopoiesis in hereditary spherocytosis with a concurrent follicular lymphoma: case report and review of the literature

**DOI:** 10.1186/s13000-015-0394-x

**Published:** 2015-09-15

**Authors:** Ricardo Molina-Urra, Daniel Martinez, Amaia Sagasta, Ana Carrio, Xavier Setoain, Benet Nomdedeu, Elias Campo

**Affiliations:** Pathology and Cytopathology Department, Hospital Base Puerto Montt, Puerto Montt, Chile; Pathology Department, Hospital Clinic, University of Barcelona, Barcelona, Spain; Nuclear Medicine Department, Hospital Clínic, University of Barcelona, Barcelona, Spain; Hematology, Hospital Clinic, University of Barcelona, Barcelona, Spain

## Abstract

We report an unusual case of a 70-year-old male with history of hereditary spherocytosis (HS) and secondary paraspinal extramedullary hematopoiesis with a concurrent follicular lymphoma. The lesion presented as a thoracic paraspinal mass of 9 cm, extending longitudinally between T6 and T9 vertebral bodies. Incisional biopsy revealed that this mass included mature hematopoietic tissue compatible with extramedullary hematopoiesis (EMH). The tissue also presented an extensive and diffuse infiltration by an atypical lymphoid population composed predominantly by small cells. The immunohistochemical study revealed that the atypical lymphoid population had a germinal center phenotype, consistent with the diffuse variant of follicular lymphoma (FL). The simultaneous presence of both EMH and FL in the same lesion made the interpretation and the final diagnosis of this case difficult. The presence of EMH in this clinical context may eclipse the diagnosis of the underlying lymphoproliferative neoplasm. The close association between the tumor cells and extramedullary hematopoietic tissue in the absence of lymphadenopathies or other tissue involvement suggests a relationship of this tumor with the recently described primary FL of the bone marrow.

## Background

Extramedullary hematopoiesis (EMH) defined by the presence of hematopoietic tissue outside the bone marrow, is a physiological phenomenon in the liver during the normal fetal development, but it is not normal after birth and must be considered a pathological finding [1]. EMH frequently affects the spleen and liver, usually forming small aggregates diffusely located in the parenchyma, but it can also appear as a pseudotumoral mass, especially in atypical locations. This lesion can be symptomatic, presenting systemic and/or local symptoms by compression of adjacent structures [1, 2]. Pathologic EMH is usually associated with other hematologic disorders affecting the bone marrow or peripheral blood. The most frequent underlying diseases associated with EMH are some chronic myeloproliferative neoplasms, chronic lymphoproliferative disorders, chronic hemolytic and inherited anemias, such as thalassemia, sickle cell disease or hereditary spherocytosis (HS) [3, 4].

Hereditary spherocytosis is an inherited form of chronic hemolytic anemia secondary to deficit of some of the cytoskeletal proteins associated with the erythrocyte membrane (Band 3, Ankyrin 1, 4.2 protein, alpha or beta Spectrin). The usual findings in this disease are the presence of spherocytes in peripheral blood, signs of chronic hemolytic anemia and splenomegaly. Eventually it could be associated with EMH in typical or atypical sites [3, 4].

The association between EMH and a concurrent lymphoproliferative disorder is uncommon. The pseudotumoral presentation of EMH and the difficulty to recognize the underlying lymphoid neoplasm hinders the diagnosis, therefore delaying the correct management of these patients. Here we present a patient with tumoral EMH associated with a follicular lymphoma and a review the literature of this uncommon association.

## Case report

**Fig. 1:**
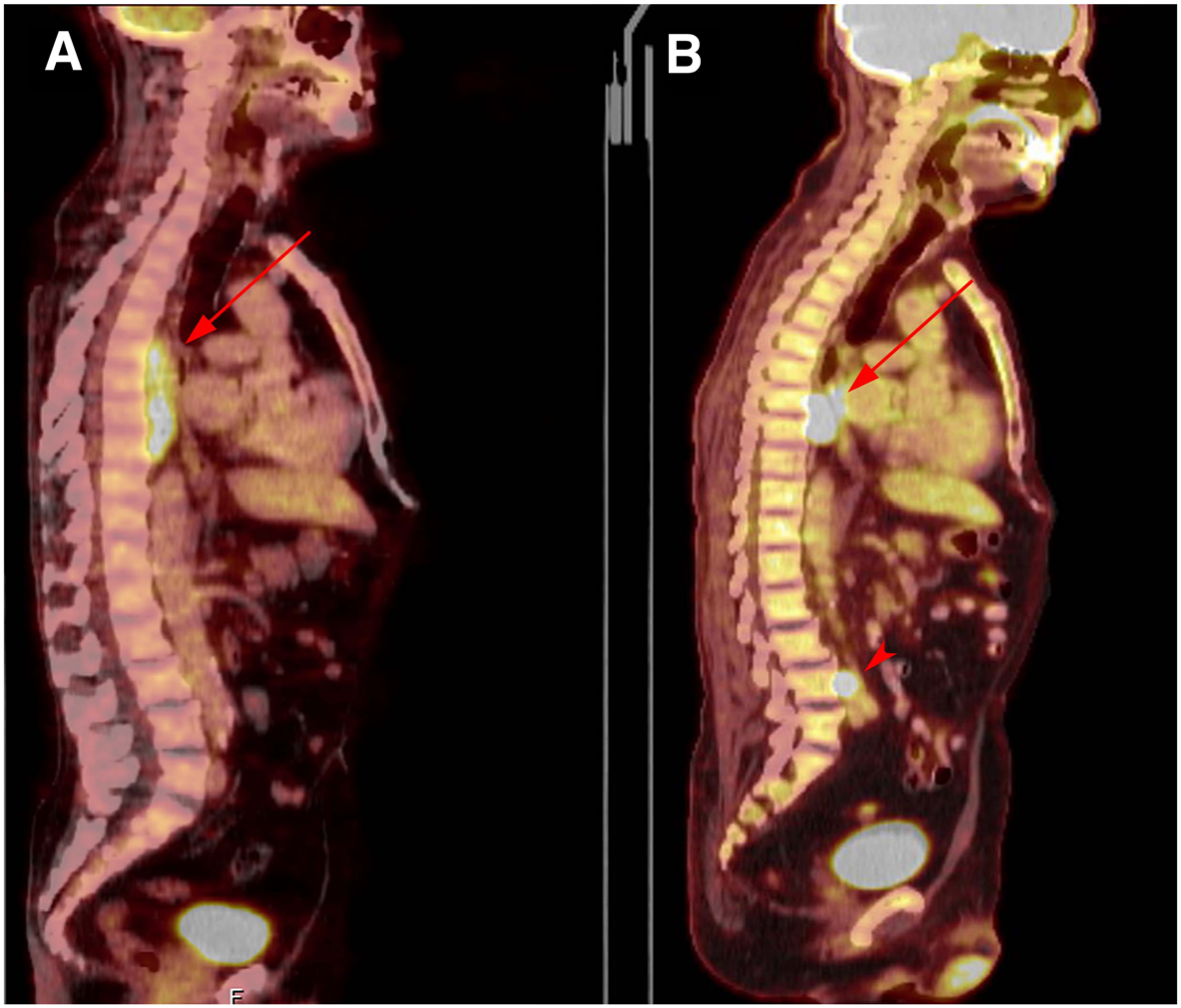
PET scan imaging before and after four weeks of Rituximab. **a** PET/CT scan before immunotherapy. Note a large metabolically active paravertebral mass of soft tissues adjacent to thoracic vertebral bodies T6 to T9 (arrow). A lesion in inter-aorto-caval mass is not visible at this level. **b** PET scan after immunotherapy; partial uptake reduction of the main thoracic paravertebral mass (arrow) and the inter-aorto-caval lesion (arrowhead)

A 70 y-o male was admitted to the hospital because of a large paravertebral tumoral mass. The patient had history of HS with mild clinical signs of hemolysis, such as pallor, jaundice and splenomegaly. Laboratory tests were consistent with underlying chronic hemolysis, with hemoglobin levels around 10–11 g/dl, reticulocyte count of approximately 5 %, ferritin levels between 376 and 470 ng/ml and transferrin saturation around 40 to 60 %. Routine abdominal ultrasonography showed splenomegaly and biliary sludge in gallbladder. The patient was in treatment with folic acid. Due to an episode of a lower respiratory infection, a CT scan and MRI were performed, evidencing a thoracic paraspinal tumoral mass of 9 × 4 × 2 cm, that extended along T6 to T9 vertebral bodies and an inter-aorta-caval mass of 2.5 × 1.6 cm with similar characteristics. A PET-CT showed a pathological uptake in both paravertebral tumors, but also revealed several lesions in right humerus, right femur and L3 vertebral body (Fig 1a). With these findings, an incisional biopsy of the main paravertebral mass, a bone marrow trephine biopsy and a study of peripheral blood were performed.

### Pathological findings

Representative sections were routinely processed in buffered formalin and embedded in paraffin, according to standard procedures. Biopsy of the main paraspinal mass revealed the presence of fragments of fibroconnective and adipose tissue. In this tissue, we identified aggregates of mature hematopoietic tissue composed of megakaryocytes without atypical features, a high proportion of erythroid elements forming erythroid islands and a small number of myeloid cells, with predominance of mature forms. Dysplastic changes of megakaryocytes, aggregates of megakaryocytes, left shifted maturation or an increase in number of blast cells were absent. These findings were consistent with extramedullary hematopoiesis. In other areas of the same sample, the tissue was diffusely infiltrated by an atypical lymphoid population of mainly small cells with irregular, indented, hypercromatic nuclei and a few large centroblast-like cells. Scattered mitotic figures were seen.

**Fig. 2:**
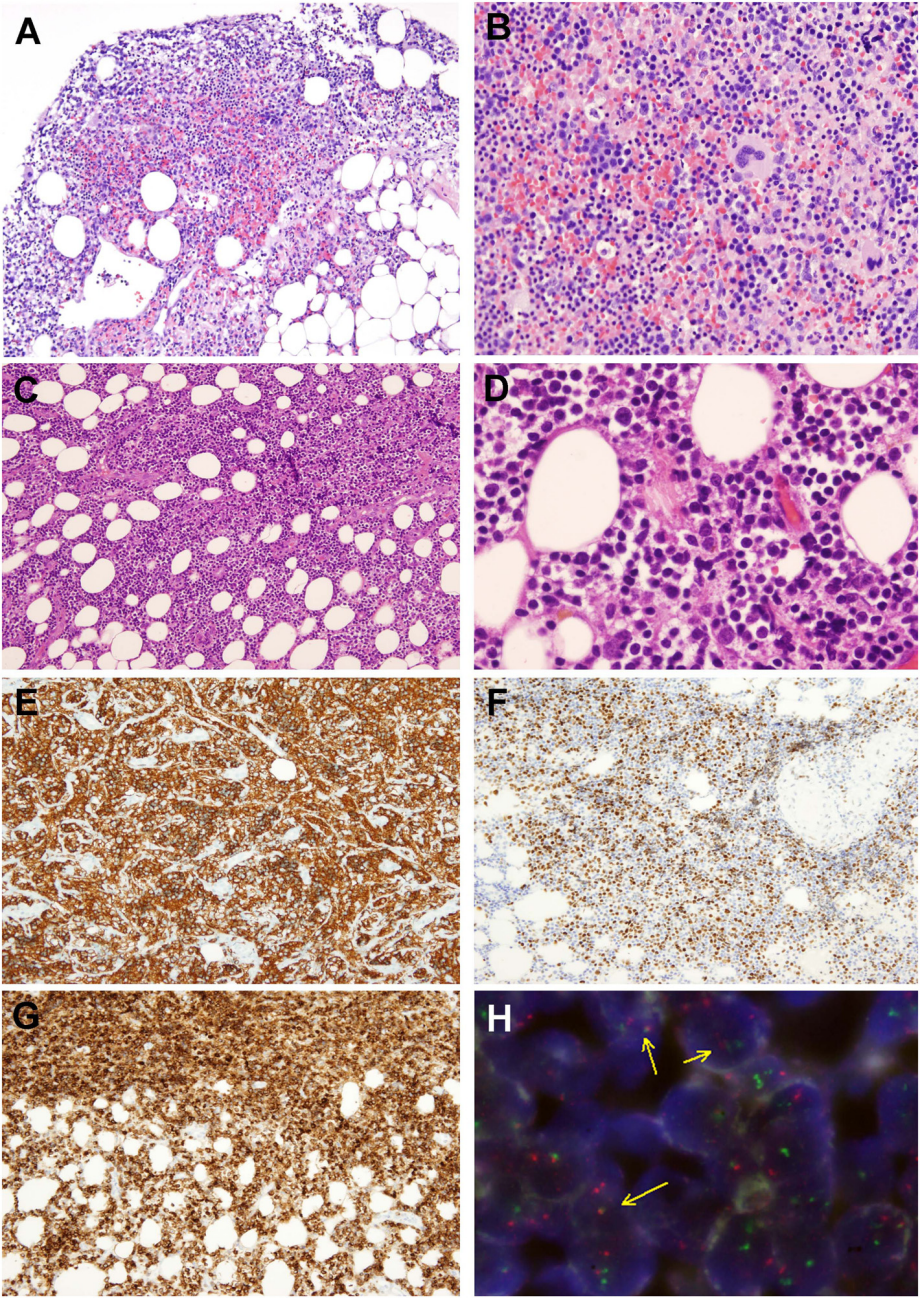
The biopsy of the paravertebral mass showed aggregates of mature hematopoietic tissue, with erythroid islands and normal megakaryocytes consistent with EMH, (**a**) H&E 40x, (**b**) H&E 200x. In other areas the tissue was diffusely infiltrated by an atypical lymphoid population of small cells with few large centroblast-like cells, (**c**) H&E 100x, (**d**) H&E 400x. The cells are positive for (**e**) CD20, (**f**) BCL6 and (**g**) BCL2. **h** FISH shows an IGH@/BCL2 translocation in some cells (yellow arrows), (dual color dual fusion probe, x400)

The immunohistochemical stains (carried out with an automated immunostainer, Dako Autostainer, Glostrup, Denmark) showed that the atypical cells had a B-cell phenotype with positivity for CD20 and CD79a and expressed the germinal center markers CD10, BCL6 and LMO2, with coexpression of BCL2. There was a variable expression of follicular dendritic cell markers (CD21 and CD23). The atypical cells were negative for CD5, CD30 and CD138. The proliferation index assessed by the nuclear positivity for Ki-67 was low, about 20 %. A prominent component of mature T-cells was found intermingled with the atypical B-cells. These findings were consistent with a diffuse infiltration by a follicular lymphoma (FL) (Fig. 2a–g). FISH for IGH/BCL2 (Abbott Molecular, Illinois) rearrangement was carried out using dual color-dual fusion probes, and evidenced t(14;18) translocation in 15–20 % of cells (Fig. 2h). The bone marrow biopsy showed an erythroid hyperplasia and a small paratrabecular infiltration by CD20 positive cells. Additional markers were not contributory. Histologic changes suggestive of a chronic myeloproliferative neoplasm were absent.

The study of peripheral blood showed some small spherical erythrocytes with dense cytoplasm consistent with spherocytes. In addition, atypical centrocytic lymphoid cells with indented nuclei and mature chromatin were also observed. Flow cytometry analysis identified a clonal population of B-cells (80 % of the total of B-cells) with expression of lambda light chain, positive for CD19, CD20, CD22, CD79b, CD23, FMC7 and negative for CD5, CD10, CD38, and CD43.

The patient began monotherapy treatment with Rituximab (375 mg/m2) during four weeks. Six months after diagnosis, PET-CT showed reduction of the main paravertebral mass, the inter-aorta-caval mass and the lesion in the humerus. A complete disappearance of the lesions in the femur and vertebrae (Fig. 1b) was observed. Rituximab was restarted and a splenectomy was performed due to chronic hemolysis. The spleen did not show any evidence of lymphoma. Six months after splenectomy, the patient is in good clinical standing.

## Discussion

HS is a group of chronic inherited anemias in which the characteristic feature is the presence of spherocytes, defined as small round erythrocytes with a higher concentration of hemoglobin. The cause is a deficiency of proteins associated with the erythroid membrane that play a critical role in the capacity of cell deformability. The proteins related to this disease are alpha-spectrin, beta-spectrin, ankyrin 1, protein 4.2 and band 3. This deficiency results in the instability of the cellular membrane with subsequent formation of membrane vesicles and secondary reduction of the surface-to-volume ratio. The spherocytes are retained in the red pulp of the spleen and part of these cells are destroyed, while the rest survive and return to the peripheral blood with even more spherocytic features. The majority of patients present signs of hemolytic anemia, such as jaundice, pallor and variable splenomegaly. Laboratory tests show mild anemia, presence of spherocytes and reticulocytes, higher mean corpuscular hemoglobin concentration and increase of the osmotic fragility of the erythrocytes. The most frequent complication of this form of chronic hemolytic anemia is the development of bilirubin gallstones. Less frequently the patient could present with skin lesions, myopathy, cardiovascular disease, neuromuscular and SNC diseases [1, 2]. HS has been rarely described in association with neoplasms, including one diffuse large B cell lymphoma [5], two acute lymphoblastic leukemias [6, 7], one splenic B-cell lymphoma [8], one schwannoma [9] and one ganglioneuroma [10]. The case of HS and ganglioneuroma presented initially as a mass in the adrenal gland and a differential diagnosis with EMH was considered.

EMH is defined by the presence of hematopoietic tissue outside the bone marrow. This phenomenon could be normal or pathologic. Pathologic EMH is usually associated with hematologic disorders affecting the bone marrow, peripheral blood and less frequently with vascular diseases or can even appear without any other underlying disorder. Spleen and liver are typical sites for EMH [3, 4]. In the largest case series, Koch et al. found 510 biopsies reporting EMH. In 483 patients, this lesion occurred in liver and spleen. In the rest of the patients, it appeared in other sites, such as paraspinal region, lymph nodes, retroperitoneum, lung, pleura, genitourinary region and skin. The underlying associated diseases were chronic myeloproliferative neoplasms, such as primary myelofibrosis, post polycythaemic myelofibrosis or chronic myeloid leukemia, chronic lymphocytic leukemia, vascular malformations and inherited or chronic hemolytic anemias [3].

Development of extramedullary hematopoiesis in the clinical context of HS has been well documented. Paraspinal, thoracic and mediastinal regions are the most frequent sites of involvement [11–20]. Independent of the underlying disease, in some patients it may present as a pseudotumoral mass, mimicking a solid tumor, and therefore a differential diagnosis with other neoplasms must be taken into account [21–23]. A concurrent lymphoproliferative disorder and EMH in the same lesion has been described in only four case reports [24–27]. In one case [24] there was no history of a previous lymphoid neoplasm, and the development of the EMH probably was in the context of a hemorragic infarct secondary to a intravascular diffuse large B-cell lymphoma in the blood vessels of the uvea. In the other three cases the lymphoproliferative disorder was previously known and the finding of EMH and a concurrent lymphoma was made afterwords. The findings of these reports are summarized in Table 1. This infrequent association between EMH and lymphoma may underestimate the presence of an underlying atypical lymphoid infiltrate, especially when extramedullary hematopoiesis presents as a pseudotumoral lesion in patients with chronic anemia or myeloproliferative disorders.

**Table 1:** Clinical and pathologic features of EMH with concurrent lymphoproliferative neoplasm in the literature

Reference	Gender	Age (years)	Anatomic site of EMH	Previous history of lymphoma	Concurrent lymphoid disease	Last follow-up
Musolino A. et al., 2007	F	36	Central nervous system	Yes	AIDS-related central nervous system lymphoma	Dead, 20 days after starting treatment
Mudhar HS. et al., [24]	F	81	Eye (uvea)	No	Intravascular diffuse large B-cell lymphoma	Alive, short follow-up
Gupta N. et al., [26]	NS	NS	Lymph node	Yes	T-cell leukemia	Alive, 4 months of follow-up
Forest F. et al., [25]	NS	53	Skin (surgical scar)	Yes	Marginal zone B-cell lymphoma	Alive, 10 years of follow-up
Current Case	M	70	Paraspinal mass (D6 – D9)	No	Follicular lymphoma, diffuse variant	Alive, 6 months of follow-up

*F*: female; *M*: male; *NS*: Not specified

In the current case, the presence of a follicular lymphoma with a predominantly diffuse pattern demonstrated in the EMH mass, in peripheral blood and in the bone marrow, is quite remarkable. Our patient did not have lymphadenopathies or other evident lesions suspicious of FL by physical examination or imaging studies. This situation is reminiscent of the recently described primary follicular lymphoma of the bone marrow in which the tumor cells were observed in this location without evidence of lymphadenopathies or organomegalies, although three of the four patients also had circulating tumor cells in peripheral blood as was observed in our patient [28]. These four cases had a diffuse, nodular or interstitial pattern of bone marrow infiltration, always with involvement of the paratrabecular area. The restricted expansion of lymphoma cells in the bone marrow in these cases suggests that the tumor cells may have a particular tropism for the hematopoietic niche [28]. The close association between the lymphoma cells and the extramedullary hematopoiesis in our case may suggest a similar phenomenon. Three of the previously described patients were asymptomatic, while the fourth one had history of bone pain without B symptoms. One patient died three months after the diagnosis without treatment response and the rest were alive at least one year after, according to their clinical follow-up. The lack of symptoms related to the lymphoma in our patient is also similar to those patients with primary FL of the bone marrow [28].

## Conclusion

We present the case of a patient with hereditary spherocytosis and secondary development of paraspinal extramedullary hematopoiesis and concurrent primary follicular lymphoma. The diagnosis of this association is rare and the presence of tumoral EMH may underestimate the existence of an underlying lymphoma. The close association between the tumor cells and extramedullary hematopoiteic tissue suggests a relationship of this tumor with the recently described primary FL of the bone marrow.

## Consent

Written informed consent was obtained from the patient for publication of this Case Report and any accompanying images. A copy of the written consent is available for review by the Editor-in-Chief of this journal.

## Abbreviations

HS: Hereditary spherocytosis
EMH: Extramedullary hematopoiesis
FL: Follicular lymphoma
